# Complete Closed Genome Sequences of Three *Bibersteinia trehalosi* Nasopharyngeal Isolates from Cattle with Shipping Fever

**DOI:** 10.1128/genomeA.00084-14

**Published:** 2014-02-13

**Authors:** Gregory P. Harhay, D. Scott McVey, Sergey Koren, Adam M. Phillippy, Jim Bono, Dayna M. Harhay, Michael L. Clawson, Michael P. Heaton, Carol G. Chitko-McKown, Jonas Korlach, Timothy P. L. Smith

**Affiliations:** a U.S. Department of Agriculture, ARS, U.S. Meat Animal Research Center (USMARC), Clay Center, Nebraska, USA; b U.S. Department of Agriculture, ARS, Center for Grain and Animal Health Research, Manhattan, Kansas, USA; c National Biodefense Analysis and Countermeasures Center, Frederick, Maryland, USA; d Pacific Biosciences, Menlo Park, California, USA

## Abstract

*Bibersteinia trehalosi* is a respiratory pathogen affecting cattle and related ruminants worldwide. *B. trehalosi* is closely related to *Mannheimia haemolytica* and is often associated with bovine respiratory disease complex (BRDC), a polymicrobial multifactorial disease. We present three complete closed genome sequences of this species generated using an automated assembly pipeline.

## GENOME ANNOUNCEMENT

*Bibersteinia trehalosi* is a Gram-negative rod bacterium that is often associated with severe acute hemorrhagic fibrinonecrotic bronchopneumonia in feedlot cattle (1, 2), sheep (3, 4), and goats (5). *B. trehalosi* and *Mannheimia haemolytica* are often distinguished only by differences in trehalose fermentation. The first *B. trehalosi* genome sequence was of USDA-ARS-USMARC-192 (GenBank accession no. CP003745) (6), a nasopharyngeal isolate that harbors antibiotic resistance cassettes, mobile elements, and other determinants that may enhance its virulence. Here, we report three additional complete closed genome sequences of *B. trehalosi* clinical cattle nasopharyngeal isolates for comparison.

Genomic DNA of *B. trehalosi* USDA-ARS-USMARC-188, -189, and -190 was extracted using a blood and cell culture DNA kit (Qiagen, Valencia CA). Sequencing was performed on a Pacific Biosciences (PacBio) RS instrument (Pacific Biosciences, Menlo Park, CA) using libraries prepared with the manufacturer’s kits with C1 chemistry. For USDA-ARS-USMARC-189, 270,000 shotgun and 127,000 paired-end Roche 454 Titanium reads were used to error correct the PacBio long reads using a hybrid error correction, as previously described (7). USDA-ARS-USMARC-188 and -190 PacBio long reads were error corrected with PBcR (6). The error-corrected read coverages for the three genomes varied from 14- to 28-fold, while the minimum read length for each genome was 6 kb. The reads were assembled using Celera assembler version 7 (6), which produced a single large contig for each isolate that was then validated and improved using Quiver (8). For all isolates, a self-self dot plot of the consensus sequences revealed at least 3.9-kb overlap between the ends of the contig at >99% identity, consistent with a circular chromosome. Duplicated sequence was removed from the 3′ end of each isolate to generate the proper circularized sequence. The origin of replication was approximated using GenSkew (http://genskew.csb.univie.ac.at), and a new linear model of the chromosome was generated using this origin position as base 1. The validity of the circularization was verified by mapping all the raw PacBio reads to this final model (including across the junction where circularization had been enforced) using Quiver, which also resolves remaining sequence errors to generate assemblies with >99.9% accuracy. A local instance of Do-It-Yourself Annotator (DIYA) (9) was used to annotate the circularized chromosome.

The *B. trehalosi* USDA-ARS-USMARC-188, -189, and -190 genome sizes are 2,340,975, 2,454,127, and 2,443,169, with gene counts of 2,221, 2,448, and 2,377, coding sequence (CDS) counts of 2,146, 2,373, and 2,301, tRNA counts of 56, 56, and 57, rRNA counts of 19, 19, and 19, and G+C contents of 38.3%, 41.0%, and 36.8%, respectively.

### Nucleotide sequence accession numbers.

The GenBank nucleotide sequence accession numbers for USDA-ARS-USMARC-188, -189, and -190 are CP006954, CP006955, and CP006956, respectively.
